# Network Adaptation Improves Temporal Representation of Naturalistic Stimuli in *Drosophila* Eye: II Mechanisms

**DOI:** 10.1371/journal.pone.0004306

**Published:** 2009-01-30

**Authors:** Anton Nikolaev, Lei Zheng, Trevor J. Wardill, Cahir J. O'Kane, Gonzalo G. de Polavieja, Mikko Juusola

**Affiliations:** 1 Department of Biomedical Science, University of Sheffield, Sheffield, United Kingdom; 2 Department of Genetics, University of Cambridge, Cambridge, United Kingdom; 3 Department of Theoretical Physics, Universidad Autónoma de Madrid, Madrid, Spain; 4 Instituto ‘Nicolás Cabrera’ de Física de Materiales, Universidad Autónoma de Madrid, Madrid, Spain; 5 State Key Laboratory of Cognitive Neuroscience, Beijing Normal University, Beijing, China; University of Southern California, United States of America

## Abstract

Retinal networks must adapt constantly to best present the ever changing visual world to the brain. Here we test the hypothesis that adaptation is a result of different mechanisms at several synaptic connections within the network. In a companion paper (Part I), we showed that adaptation in the photoreceptors (R1–R6) and large monopolar cells (LMC) of the *Drosophila* eye improves sensitivity to under-represented signals in seconds by enhancing both the amplitude and frequency distribution of LMCs' voltage responses to repeated naturalistic contrast series. In this paper, we show that such adaptation needs both the light-mediated conductance and feedback-mediated synaptic conductance. A faulty feedforward pathway in histamine receptor mutant flies speeds up the LMC output, mimicking extreme light adaptation. A faulty feedback pathway from L2 LMCs to photoreceptors slows down the LMC output, mimicking dark adaptation. These results underline the importance of network adaptation for efficient coding, and as a mechanism for selectively regulating the size and speed of signals in neurons. We suggest that concert action of many different mechanisms and neural connections are responsible for adaptation to visual stimuli. Further, our results demonstrate the need for detailed circuit reconstructions like that of the *Drosophila* lamina, to understand how networks process information.

## Introduction

**Figure 1:**
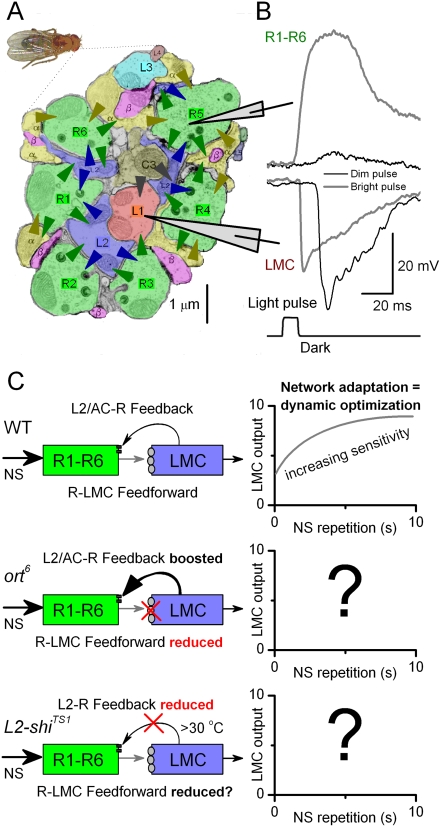
Feedforward and feedback connections in the lamina are complex. A. Color-coded electron-micrograph shows a cross-section of the neurons and synapses in a single lamina cartridge of the *Drosophila* optic lobe. R1–R6 are terminals of photoreceptors. Monopolar cells, L1 and L2, are situated in the centre of the cartridge together with a terminal of a C3-cell from the 2^nd^ visual neuropile, the medulla. Other monopolar cells, L3–L5 lie more peripherally. C2 is a second medulla cell, whereas α indicates an amacrine cell (AC) and β indicate a T1 cell. Only L1–3 and AC receive direct inputs from R1–R6 (green arrows). L4 receive inputs from AC and L4, L5 from AC and Tan (not shown), and T1 from AC [1]. L2 (blue arrows) and AC (brown arrows) are the only direct feedbacks to R1–R6 terminals [1]. Higher-order feedback to the lamina from C2 and C3 fibers is indicated by gray arrows. The figure is based on a modified EM image from Ian Meinertzhagen (http://flybrain.neurobio.arizona.edu/). The arrowheads highlight only some of the complex interactions between the neurons in this network. B. *In vivo* intracellular recordings from intact Oregon-R *Drosophila* at 25°C. A R1–R6 photoreceptor depolarizes (due to opening of light-gated cation-channels, trp(l)) [36] and a LMC hyperpolarizes (due to opening of histamine-gated Cl-channels hclA) [9] to light pulses, generating complex response waveforms. Here the saturating bright pulse is 1,000 times more intense than the dim pulse, yet these cells can reliably respond to both of them. The LMC's voltage response to the dim pulse is larger than its response to the bright pulse, in contrast to the photoreceptor output. Note that the responses of LMCs reach their peak well before those of photoreceptors. C. A block-diagram representing the feedforward (R-LMC) and direct feedback (L2/AC-R) pathways in which visual information flows in the R-LMC-R system. In this paper, we perturbate these connections to work out whether they play a role in improving the neural representation in the LMC output to repeated naturalistic contrast patterns [4].

*Drosophila*, with its well-defined genetics, modular eye structure, fully characterized synaptic layout of the first visual neuropil [1], and accessibility for intracellular electrophysiology of single neurons [2]–[4], is a powerful model for studying neural adaptation *in vivo*. The first visual neuropile, the lamina (Fig. 1A), contains a system of neurons consisting of photoreceptors (R1–R6) and interneurons, these being large monopolar cells (LMCs: L1–L3) [5], [6] and an amacrine cell (AC) that co-process visual information [7]. While photoreceptors depolarize and LMCs hyperpolarize to light (Fig. 1B), their graded voltage responses are shaped together [3].

Photoreceptors project histaminergic synaptic terminals [8], [9] onto LMCs and an AC, whilst L2 and the AC in turn feed back onto photoreceptor terminals via ligand-gated synapses (Fig. 1C). The inhibitory feedforward pathway (R1–R6→LMCs/AC, henceforth simplified as R-LMC) [3], [8]–[10] uses the same hclA histamine-receptor, sometimes named ort [10], whereas the feedback pathway (L2/AC→R1–R6, henceforth simplified as LMC-R) is excitatory [3], [11], [12]. This circuitry results in negative feedback via the reduction of excitation, which is a different mechanism from direct feedback inhibition [3], [13]. This simplified view of processing, designated as R-LMC-R system, leaves out cells and synapses in the laminal circuit that receive indirect R1–R6 input (Fig. 1A), these being C2–C3 fibers, L4–L5, T1 and Tan cells. The simplified R-LMC-R model also ignores glia, which receives R1–R6 input through a different histamine-receptor, hclB [1], [10]. The roles of these 2^nd^- or 3^rd^-order connections and glia in visual processing are still largely unknown.

In the steady state, because of the tonic release of neurotransmitter [3], [14], the membrane potential of photoreceptors is a function of (i) light-mediated conductance and (ii) synaptic conductance, mediated by L2 and AC negative feedback. One outcome of this negative feedback can be to keep the photoreceptor membrane potential largely independent of the light intensity, thus avoiding saturation [3]. When light intensity is varied over time, the photoreceptor membrane potential is the result of the dynamic interaction of the *changes* in the light-mediated and the feedback-mediated synaptic conductances [3]. Because of the different time course of these conductance changes, complex responses occur, including boosting of the photoreceptor response to the onset of a light pulse [3].

In a companion paper [4], we have shown the relevance of adaptation for efficient coding through in the R-LMC-R system. Here we use these results to find the neural mechanisms for enhancing the LMC output (Fig. 1C) to repeated naturalistic light stimuli. We again use the same naturalistic stimulation protocol to study adaptation on behaviorally relevant time scales [4]. By comparing the outputs of wild-type photoreceptors and LMCs to those in which either the feedforward (R-LMC) or feedback (L2-R) pathways are severely compromised, we show that the enhancement of the LMC output via network adaptation in the *Drosophila* eye needs both of these pathways. These findings suggest that neural information processing is very distributed even at the very first stage, the layer of first visual synapses.

## Results

Results of our companion paper [4] suggested that the dynamic improvement in the LMC output to repeated naturalistic stimulus probably reflected a gradual increase in histamine-gated chloride conductance [9]. There are two mechanisms that could modify this “input conductance” in LMCs: (i) an increase in the open-probability of postsynaptic histamine receptors, or (ii) an increase in presynaptic histamine release probability, *i.e.* ligand concentration. It is likely that these mechanisms are continuously reset by dynamic equilibrium between light- and feedback-mediated conductances. We test these hypotheses by separately manipulating the strength of the synaptic feedforward (R-LMC) or feedback (L2-R) pathways.

### Testing the feedforward pathway: *ort^6^* mutant lacks adaptation

**Figure 2:**
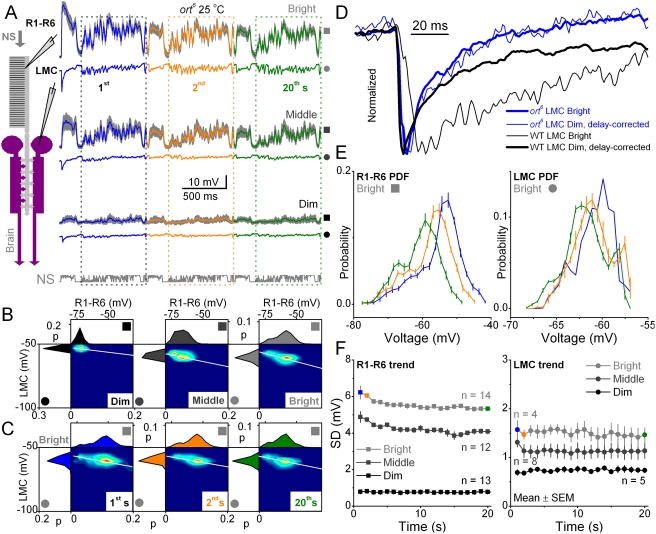
The *ort^6^* R-LMC-R system show limited adaptation to naturalistic stimuli (NS). Throughput of the feedforward pathway from R1–R6 photoreceptors to LMCs is compromised by ort^6^ receptors, which reduce histamine sensitivity by >10-fold [8]. A. Voltage responses of *ort^6^* photoreceptors (mean±SD, gray, n = 7) and a representative LMC to a second long naturalistic stimulus (NS), during the first stimulus repetitions at different luminance levels at 25°C. B. The corresponding probability density functions (PDFs) for R1–R6s (top) and LMCs (left) and the joint probability density functions of the first 20 responses. In contour plots of jPDFs, hot colors denote high probability. The jPDFs quantify the input-output transformations, characterizing the synaptic gain for the given luminance of stimulation. The most probable synaptic gain, *i.e.* the slopes of the white lines, is quite invariable. C. The photoreceptor and LMC PDFs and jPDFs shown at the 1^st^, 2^nd^ and 20^th^ s of bright stimulation. Note that the synaptic gain (white lines) varies little over time unlike in the WT system [4]. D. The normalized responses of *ort^6^* (blue) and WT Oregon-R LMCs (black) to dim (thin traces) and bright stimuli (thick traces). Ort^6^ receptors mimic extreme “light adaptation”, making the speed (time course) of the LMC output invariable at different intensities. E. High resolution PDFs of photoreceptor (left) and LMC (right) outputs shown at different times during bright stimulation. The PDFs of *ort^6^* photoreceptors show a gradual compression in voltage range, whilst the PDFs of *ort^6^* LMCs remain unchanged after the first second of stimulation. F. The SDs of voltage responses indicate that the adaptive trends in the photoreceptor or LMC outputs are quite similar for each luminance level. Thus, sensitivity of these cells either remains the same or reduces gradually during repetitive stimulation, unlike in WT LMCs, in which the sensitivity increases over stimulation [4]. WT LMC output has an increasing trend, similar to Fig. 1C. SDs are from the boxed data (201–1000 ms) in A.

The feedforward pathway can be perturbed by faulty transmitter receptors on the LMC membrane. Hence, to test the contribution of the histamine receptors to the observed adaptation dynamics in the R-LMC-R system, we recorded from photoreceptors and LMCs in *ort^6^* mutants (Fig. 2). These flies have desensitized histamine receptors in LMCs and AC [3], [8], [10]. Figure 2A shows the first, second and twentieth second in the photoreceptor and LMC output of *ort^6^* flies in response to dim, middle-intensity and bright stimulation. The voltage responses of *ort^6^* photoreceptors were larger and faster than those of wild type (WT) photoreceptors, whereas the responses of *ort^6^* LMCs were always smaller than in WT flies receiving similar stimulation. These comparisons are not shown here, but see [3], [4]. We have recently shown evidence that reduced sensitivity of *ort^6^* receptors depolarizes LMCs and possibly ACs, and that this artificially high membrane potential enhances their excitatory synaptic feedbacks to the photoreceptor terminals [3]. Accordingly, the voltage responses of *ort^6^* photoreceptors are boosted [3]. The larger voltage responses of *ort^6^* photoreceptors could then enhance voltage-sensitive histamine release [3], [14] from photoreceptor terminals. In agreement with this scheme, the amplitude range of *ort^6^* LMCs increased as a function of light intensity, reaching at bright stimulation a similar range to that of WT LMCs at dim stimulation. See also Fig. 2A in [4].

### *Ort^6^* receptors cannot boost LMC output to repeated naturalistic stimulation

*Ort^6^* synapses showed very limited gain adjustments with light intensity, as judged by the slopes in the joint probabilities of photoreceptor and LMC outputs at dim, middle and bright stimulation (Fig. 2B, white lines). There were also no obvious changes in gain over time (Fig. 2C). Consequently, there were no clear differences in adaptation with light intensity, as depicted in Figure 2D that shows the normalized changes for WT and *ort^6^* LMCs. While WT LMCs had different adaptational properties under dim and bright light, *ort^6^* LMCs showed no difference. This inability to change the dynamics over a time scale of seconds was apparent in the absence of broadening of the probability distributions of LMCs, as seen in Figure 2E, which showed only a small shift but no change of distribution width. Thus, it seems that unlike WT receptors, *ort^6^* receptors lack a mechanism for the time- or dose-dependent increase of LMC output during stimulation, as indicated by their flat or decreasing sensitivity over time (Fig. 2F).

**Figure 3:**
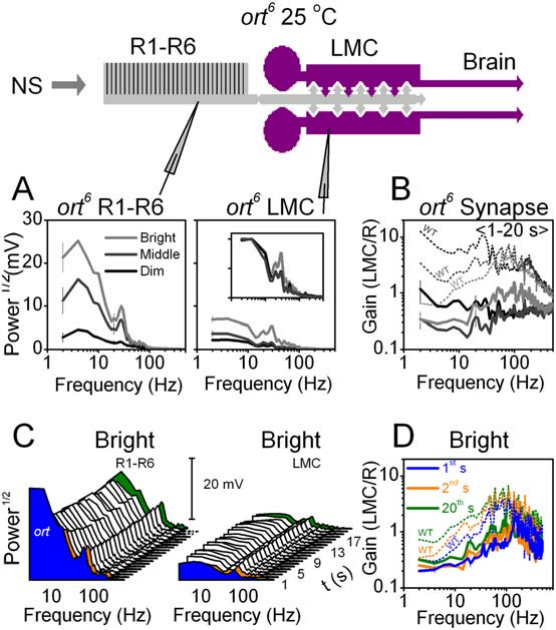
The *ort^6^* R-LMC-R system lacks adaptational enhancement of low frequency signals. A. Mean frequency spectra of seven *ort^6^* photoreceptors (left) and a representative LMC (right) to dim, middle and bright naturalistic stimuli (NS). Inset shows the normalized LMC frequency spectra. B. Synaptic gain is the highest with bright stimulation. Corresponding WT synaptic gain (dotted lines) is given for comparison, from [4]. C. Changes in *ort^6^* photoreceptor (left) and LMC (right) frequency spectra for 20 seconds of repeated bright stimulation. The 1^st^ (black), 2^nd^ (red) and 20^th^ (green) frequency spectra, respectively. D. The frequency range of *ort^6^* synaptic gain, dominated by high frequencies, spreads only marginally over time in comparison to the average WT Oregon-R synaptic gain (dotted lines) for the same experiment [4]. Error bars are SDs.

What mechanism could cause such differences in the dynamics of WT and *ort^6^* receptors? The simplest explanation is that with their reduced histamine sensitivity, *ort^6^* receptors also have a reduced ability to remove desensitization (deactivation). As the time course of this process is slower than the activation of histamine-gated channels [8], it should primarily affect the transmission of low frequency signals. For corroboration, we analyzed the frequency spectra of *ort^6^* photoreceptors and LMCs. These showed the expected light- (Fig. 3A) and time-dependencies (Fig. 3C). The enhanced feedback of *ort^6^* mutants [3] caused the photoreceptor output to follow higher stimulus frequencies than in WT flies [4], even up to 60–70 Hz, but otherwise they adapted in a similar manner. On the other hand, *ort^6^* LMC output had a limited increase in frequency with stimulus repetition compared to WT [4]. As suggested, *ort^6^* LMC output showed a particularly weak representation of low frequencies, failing to follow slow changes in histamine concentration. The synaptic gain (Figs. 3B) was low compared to WT (dotted lines). Concerning the dynamics of frequency representation, there was a slight increase in the low and middle frequencies (Figs. 3D), but this was small compared to WT flies (dotted lines).

### Testing the L2-R feedback pathway: *L2-shi* ^TS1^ lacks adaptation at 30°C

The L2-R feedback should exert network adaptation to R1–R6 photoreceptors by modulating their output [3]. Hence, to test whether L2-R feedback affects the dynamics of histamine release from photoreceptors and the observed adaptation dynamics of photoreceptor and LMC output, we recorded from photoreceptors and LMCs in temperature-sensitive *L2-shi^TS1^* flies. In these flies [15], *shibire^TS1^* reversibly reduces signaling in L2-R connections at 28–30°C, *cf.* [3], [15], [16]. We can, thus, effectively switched off and on synaptic feedback from L2-monopolar cells to R1–R6 photoreceptor terminals by warming and cooling the flies to restrictive (30°C) or permissive (19°C) temperatures, respectively. Because the rate of transmitter release depends on presynaptic voltage, removing the excitatory L2 feed to photoreceptors should lower their voltage level [3], and therefore reduce histamine release. This, we hypothesize, should then reduce adaptability in the synaptic signal transfer from R1–R6 to LMCs.

**Figure 4:**
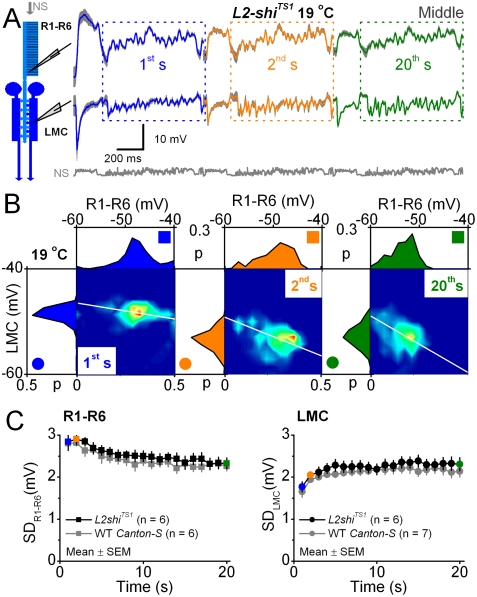
Adaptation happens normally in the *L2-shi^TS1^* R-LMC-R system at 19°C. At this permissive temperature, photoreceptor and LMC outputs to a repeated naturalistic stimulation (NS) in *L2-shi^TS1^* cells are similar to the corresponding outputs of WT Canton-S cells. A. The 1^st^, 2^nd^ and 20^th^ voltage responses of R1–R6 photoreceptors (mean±SD, gray, n = 6, above) and LMCs (mean±SD, gray, n = 6, below) to middle intensity stimulation. B. The corresponding photoreceptor and LMC PDFs and jPDFs during bright stimulation. The most probable synaptic gain (the slope of white lines) increases over time, similar to WT [4]. C. The adaptive trends of photoreceptor (left) and LMC (right) output, measured as SD the boxed data in A (201–1000 ms) to repeated stimulation. Adaptation dynamics are similar in the *L2-shi^TS1^* (black) and WT (gray) R-LMC-R systems.

**Figure 5:**
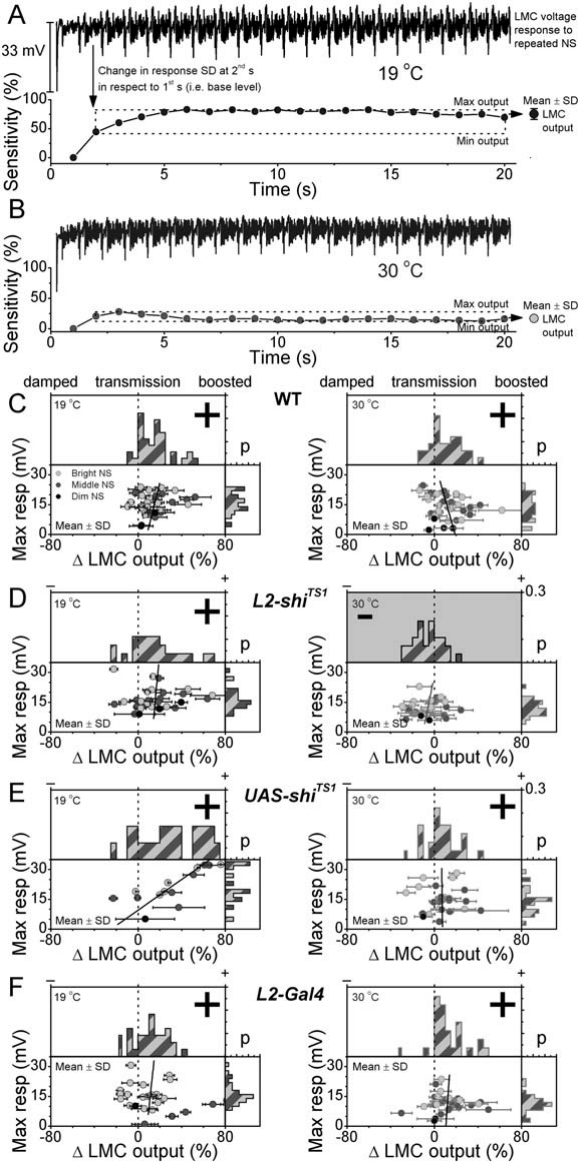
Genetic controls shows that adaptation in LMC output is dampened by reducing L2-R feedback. Statistical characterization of adaptive trends is for 4-days-old *L2-shi^TS1^*, WT and control flies (*UAS-shi^TS1^*; *L2-Gal4*), all having Canton-S (*CS*) red genetic association. The recordings are at 19°C (blue) and 30°C (orange). A. Typical high-quality voltage response of a LMC to a bright repetitive naturalistic stimulation (NS) at 19°C. This data is from a *UAS-shi^TS1^* fly. Sensitivity, *i.e.* change in LMC output (gray circles) is defined as the mean of the standard deviations of consecutive response segments to a second long naturalistic stimulus (NS) pattern, from the 2^nd^ to 20^th^ s (dotted box, each data point is calculated from 800-ms data sections as in Fig. 2). This change is then plotted as a percentage, in respect to the 1^st^ response, showing 75% increase in the LMC output in 20 seconds. B. Voltage response of the same LMC, but at 30°C. Notice the faster adapting trend. LMC output increases 30% in 3 seconds before settling to 10%, due to faster bioreactions at the higher temperature [18]. Accordingly, the voltage responses at 30°C are often smaller than at 19°C. C–F. Changes in the mean LMC output of all recordings (with adequate stability) plotted against their maximum responses at 19°C (left) and at 30°C (right) for different flies as probability distributions. The left hemi-field indicates decaying and the right increasing LMC output. The fits show the mean correspondence between the adapting trends and maximum responses. At 19°C, the fits lie to the right, *i.e.* the larger the response the larger is its adaptational boosting over the duration of the recording. C. WT Canton-S LMC output is boosted in 33/36 recordings (92%) at 19°C and 27/34 recordings (79%) at 30°C. D. *L2-shi^TS1^* LMC output is boosted in 21/27 recordings (78%) at 19°C, but only 6/29 recordings (21%) show a small increase at 30°C (when L2-R-feedback is reduced). These population means differ significantly (p = 0.00004, ANOVA, one-way Bonferoni test). Thus, LMC output of *L2-shi^TS1^* decreases at 30°C (gray highlight), in contrast to all other genotypes that show an increase over time. E. *UAS-shi^TS1^* LMC output is boosted 11/14 recordings (78%) at 19°C and 20/27 recording (74%). F. *GAL4-L2* LMC output is boosted in 25/33 recordings (76%) at 19°C and 25/27 recordings (93%) at 30°C.

We first confirmed that synaptic throughput, and thus adaptation to repeated stimulation, was normal in the *L2-shi^TS1^* R-LMC-R system at permissive temperature. Figure 4A shows the first, second and twentieth voltage responses of their photoreceptors and LMCs to the middle-intensity stimulus. Adaptation was the same as for WT flies; the spread of responses narrowed in photoreceptors (Fig. 4B; x-axis) and widened in LMCs (y-axis) with the most probable synaptic gain (white lines) increasing with time. Consequently, the distinctive adaptive trends, *i.e.* the standard deviation (SD) in the photoreceptor or LMC outputs over time (Fig. 4C), were similar in both flies. With L2-R feedback operating, the LMC output was boosted over stimulus repetitions, *cf.* [4]. Adaptation in the other control flies was also similar (Fig. 5A–F).

**Figure 6:**
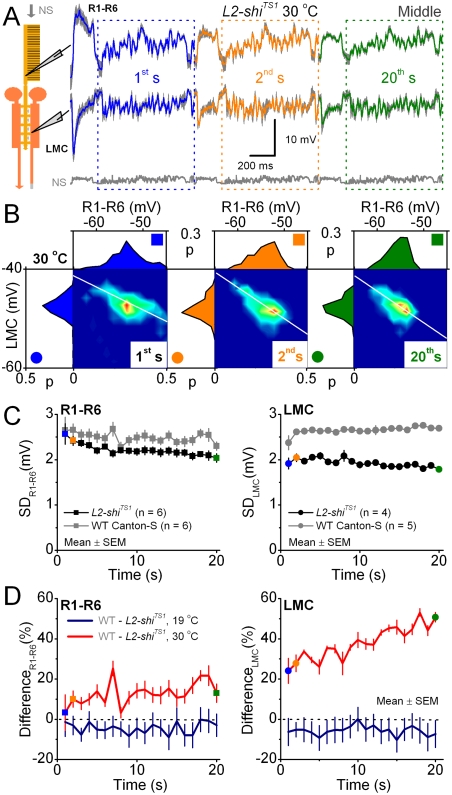
Adaptation in the *L2-shi^TS1^* R-LMC-R system is reduced at 30°C. At this restrictive temperature, *Shibire^TS1^*-proteins should block endocytosis [16] in L2s, reducing their feedback to R1–R6 photoreceptors in *L2-shi^TS1^* flies. A. The 1^st^, 2^nd^ and 20^th^ voltage responses of R1–R6 photoreceptors (mean±SD, gray, n = 6, above) and LMCs (mean±SD, gray, n = 4, below) to middle intensity naturalistic stimulation (NS) at 30°C (light yellow panels). The responses are somewhat smaller than at 19°C, *cf.* Fig. 4A. B. The corresponding probability density functions, PDFs, and joint probability density functions, jPDFs, during bright stimulation at 30°C. The most probable synaptic gain (white lines) remains invariable over time. C. SD of photoreceptor (left) and LMC (right) output at different times to repeated stimulation at 30°C. The *L2-shi^TS1^* photoreceptor and LMC outputs lack the adaptational boosting of WT controls (gray). D. The difference: 100*[WT(output)-*L2-shi^TS1^*(output)]/ *L2-shi^TS1^*(output) in restrictive temperature (30°C, red) is calculated for 19°C and 30°C data separately. At 30°C, L2-R feedback in *L2-shi^TS1^* flies is reduced, but WT flies have functional L2-R feedback. The difference in the corresponding LMC outputs shows that L2-feedback boosts WT LMC output to repeated stimulation by ∼40%. L2-feedback seems also to boost WT R1–R6 output by ∼15%. The control experiments at 19°C show that L2-feedback functions normally in both *L2-shi^TS1^* and WT cells, with only little differences in their relative strengths (*cf.*, Fig. 4).

In contrast, at restrictive temperatures, LMC output decreased in *L2-shi^TS1^* flies but not in control flies (Figs. 6 and 5A–F). Recordings were performed after warming the flies for over five minutes, as this was likely to deplete the synaptic vesicles in *shi^TS1^* mutants [3]. In this situation, LMCs continued to receive histaminergic input from photoreceptors (Fig. 6A). However, responses of both photoreceptors and LMCs appeared smaller than at 19°C, and their adaptation dynamics were clearly reduced (Figs. 6B and 5D). With continuing stimulation, the spread of both the photoreceptor (x-axis) and LMC (y-axis) outputs stayed the same or narrowed slightly, while the most probable synaptic gain (white lines) remained practically unchanged, similar to *ort^6^* flies (*cf.* Fig. 2C).

These findings imply that when L2-R feedback is reduced, both histamine release from photoreceptors and the amplification of post-synaptic signaling suffer. Hence, the adaptive trends in LMC output should now track the photoreceptor input. With the mean voltage in photoreceptor terminals falling, histamine release should slow down, reducing the high-frequency content of the LMC output, *i.e.* resembling dark adaptation [17]. These predictions were tested by comparing the mean membrane potentials of photoreceptors and LMCs and their adaptive trends in *L2-shi^TS1^* flies to those of controls at 19 and 30°C (Figs. 6C–D; Figs. 4C and S1).

Adaptation dynamics changed when L2-R feedback was reduced at 30°C. First, the voltage range of *L2-shi^TS1^* photoreceptors and LMCs shifted (data not shown). As expected, the mean potential of *L2-shi^TS1^* photoreceptors hyperpolarized in comparison to the WT photoreceptors by −3.0±0.8 mV (mean±SD, n = 6 cells; over 1–20 s, data measured in the somata), further supporting the previous findings of L2-R feedback being excitatory [3]. Likewise, *L2-shi^TS1^* LMCs were more depolarized than WT LMCs (up by 11.0±0.5 mV; mean±SD, n = 5 cells; over 1–20 s), indicating that they received less histamine (histamine hyperpolarizes LMCs). Second, the utilization of the voltage range (SD) of *L2-shi^TS1^*cells diminished over time (Fig. 6C). Here, 23 out of 29 LMC recordings (dim, middle and bright stimuli) were adaptation-deficient (right), having approximately constant or decaying trends similar to those of R1–R6 (left). The remaining six showed only a small increase (Fig. 5D). Because L1 and L2 are the largest cells in the lamina, and thus the most likely recording sites, these data must include both L1 and L2 cells. Therefore, reducing the L2-R feedback must affect the outputs of both the L1- and L2-pathways. Notice that warming naturally accelerated response dynamics (*cf.* Fig. 5A–B) presumably by speeding up biological processes [18], thus making the adapting trends in the LMC output also faster.

Because the genetic controls (Figs. 4–5) performed as WT flies, and adaptation in the WT and *L2-shi^TS1^* R-LMC-R systems occurred similarly at 19°C (Fig. 6D, blue), the differences in the responses of *L2-shi^TS1^* flies at 30°C (red) had to result from reducing L2-R feedback. Hence, we could approximate its effect on adaptation by subtracting the outputs of *L2-shi^TS1^* cells from those of the corresponding WT cells. It emerges that L2-R feedback over the first 20 s of naturalistic stimulation was necessary for sensitivity control of synaptic signal transfer, boosting the R1–R6 and LMC outputs by ∼15% and ∼40%, respectively.

### L2-R feedback participates in adaptive “whitening” of LMC output

**Figure 7:**
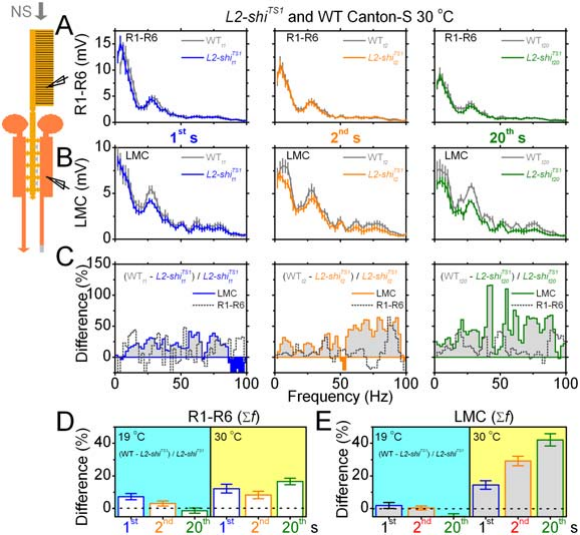
Adaptation acting through L2-R feedback helps to “whiten” LMC output. A. R1–R6 photoreceptor and B LMC mean frequency spectra for the 1^st^ (left), 2^nd^ (middle) and 20^th^ (right) voltage response of *L2-shi^TS1^* (blue, orange, green) and WT Canton-S (gray) flies at 30°C. The recordings show mean±SEM (photoreceptors: n = 7–9, LMCs: n = 5–12). C. The relative difference in the frequency spectra between WT and *L2-shi^TS1^* LMC (gray areas) and photoreceptors (transparent areas under dotted lines). L2-R feedback “whitens” and extends the frequency range of LMCs over time by boosting mostly high-frequency signals (50–100 Hz). D–E. The panels show the mean±SEM of the relative differences in C for R1–R6 and LMCs, respectively, at 19°C (white) and 30°C (gray). Gray bars differ significantly from white bars in all comparisons. L2-R feedback increases photoreceptor and LMC output by ∼15% and ∼40%, respectively, over time.

How does the L2-R feedback affect the frequency spectra of photoreceptor and LMC outputs? Figures 7A and B show these frequency spectra for the 1^st^, 2^nd^ and 20^th^ voltage responses to the stimulus in *L2-shi^TS1^* and WT flies (gray) at 30°C. The stronger WT frequency spectra indicated that L2-R feedback boosted both photoreceptor and LMC output. More specifically, the relative differences between the WT and *L2-shi^TS1^* spectra (Fig. 7C; controls in Fig. S1) showed that amplification by L2-R feedback increased with time, occurring mostly at the middle- and high-frequencies of the LMC output (gray areas).

We then compared these relative differences for the corresponding photoreceptor and LMC outputs (Figs. 7D and E, respectively) over the signaling range (2 to 100 Hz) at permissive and restrictive temperatures. We were not interested in the response frequencies higher than 100 Hz, because these mostly contain noise. At permissive temperature, the enhancement of photoreceptor and LMC outputs in the *L2-shi^TS1^* R-LMC-R system was marginally stronger than in WT (*cf.* Fig. 5C), leading to slight but insignificant decays in their output ratios over time. In contrast, at restrictive temperature, these output ratios grew significantly and progressively during stimulus repetitions with L2-R feedback boosting the WT photoreceptor and LMC outputs by ∼15% and 40%, respectively. The matching time-domain values (*cf.* Fig. 6D) confirm the frequency analysis.

### L2-R feedback improves signal-to-noise ratio of LMC output

**Figure 8:**
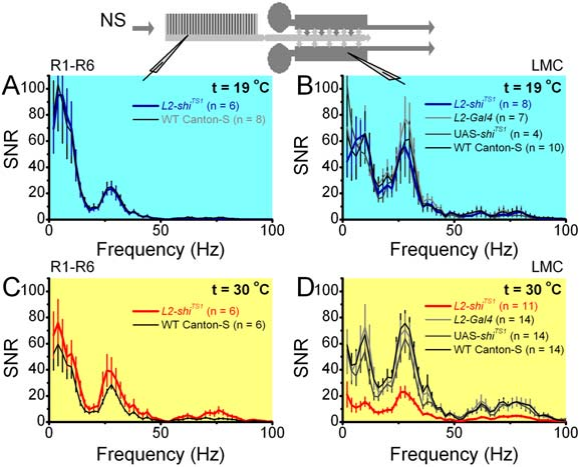
L2-R feedback improves the signal-to-noise ratio (SNR) of LMC output. A–B. The panels show the signal-to-noise ratio in *L2-shi^TS1^* (blue) and WT (black) photoreceptors and LMCs, respectively, to the same middle intensity naturalistic stimulation (NS) at 19°C (permissive temperature). C–D. The panels show the signal-to-noise ratio in *L2-shi^TS1^* (red) and WT (black) photoreceptors and LMC, respectively, to the same stimulus at 30°C (restrictive temperature). Notice that the signal-to-noise ratio of *L2-shi^TS1^* LMCs collapses at the restrictive temperature. Notice also that *L2-Gal4* and *UAS-shi^TS1^* LMCs have similar signal-to-noise ratios to WT LMCs, indicating that Gal4 and the UAS lines themselves did not influence signaling performance of the R-LMC-R system. Mean±SEM are shown, n is the number of cells in each group.

Without L2-R feedback the *L2-shi^TS1^* R-LMC-R system failed to adapt appropriately and the size and speed of both photoreceptor and LMC outputs diminished over stimulus repetition. To measure how this deficient adaptation changes their signaling performance, we calculated their signal-to-noise ratios for the *L2-shi^TS1^* and control systems at permissive and restrictive temperatures (Fig. 8). Signal-to-noise ratio estimation assumes that the system operates stationarily, and that signal and noise add linearly the response. These assumptions are not fully true for neural responses; see [19]. Signal-to-noise ratio of photoreceptors and LMCs are underestimates. The noise, *i.e.* the difference between the signal (mean response) and individual responses, includes both adaptation and stochastic variability. However, since here each signal-to-noise ratio was calculated using data sections in which the major adapting trends had already subsided (10–20^th^ s) and using the same amount of data (11×1,000-points), their means could be compared. Thus, we used signal-to-noise ratio only as an approximate measure of the impact of L2-R feedback on neural coding by comparing these estimates in the test and control R-LMC-R systems.

At permissive temperatures, the average signal-to-noise ratio of *L2-shi^TS1^* photoreceptors and LMCs matched those of controls (Fig. 8A and B, respectively). Pooling the inputs from six photoreceptors for each sampling point in a visual scene [20] boosted the signal-to-noise ratio in LMCs, broadening its frequency range over that of photoreceptors [3], [17], [21]. However, when L2-R feedback was reduced at 30°C, photoreceptor and LMC signal-to-noise ratios changed (Figs. 8C and D, respectively). There was a slight improvement in the signal-to-noise ratio of *L2-shi^TS1^* photoreceptors compared to WT, suggesting that possibly other neural feedbacks to photoreceptors (such as amacrine cells) were acting to improve the faulty synaptic signal transfer by increasing the network regulation on photoreceptor output; see [3]. Alternatively, L2-R feedback could be detrimental to the photoreceptor signal-to-noise ratio as defined in this instance. It is also possible that the role of L2-R feedback is not to maximize signal-to-noise ratio at the level of the photoreceptor. However, resolving this issue requires more focused studies in the future.

In agreement with the decline in LMC output above (*cf.* Fig. 7D), the signal-to-noise ratio of *L2-shi^TS1^* LMCs dropped to ∼1/3 of the signal-to-noise ratio of WT LMCs and other genetic controls. This drop was particularly evident in middle and high frequencies, where the responses of *L2-shi^TS1^* LMCs are proportionally diminished most (*cf.* Fig. 7C and Fig. S1D). Noticeably, the signal-to-noise ratio of *L2-shi^TS1^* LMCs was also less than that of the photoreceptors. Thus, normally functioning L2-R feedback in WT flies helps to make the voltage responses of LMCs not only larger and faster but also less noisy.

## Discussion

**Figure 9:**
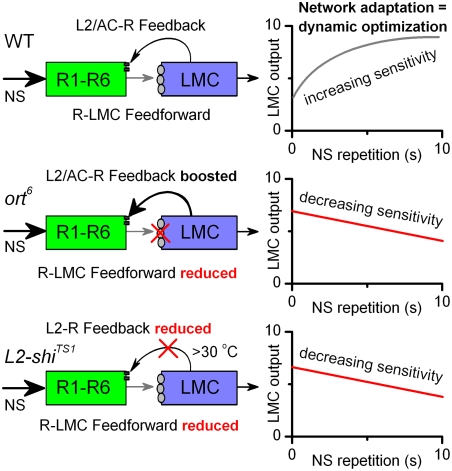
Feedforward and feedback pathways are necessary components for network adaptation. In the WT R-LMC-R system (top), LMC output increases with naturalistic stimulus (NS) repetitions by network adaptation (increasing sensitivity) set by a dynamic equilibrium between light-mediated conductance and feedback-mediated synaptic conductances. In the *ort^6^* R-LMC-R system (middle), mutated ort^6^ histamine receptors act as a strict high-pass filter [8], reducing throughput of the feedforward pathway and depolarizing LMCs and AC. This amplifies the feedback connections to photoreceptors, pushing the dynamic equilibrium toward extreme “light adaptation” in all luminance levels. Thus, LMC output contains predominantly high-frequency signals and cannot be improved over time (decreasing sensitivity). In the *L2-shi^TS1^* R-LMC-R system at 30°C (bottom), L2-R feedback is reduced and the dynamic equilibrium shifts toward dark adaptation. As less information is returning to photoreceptors, their output is modified less efficiently and the LMC output desensitizes, tracking mostly the photoreceptor adaptation (decreasing sensitivity).

In the preceding companion study [4], we showed that network adaptation in the R-LMC-R system boosts under-represented signals in the LMC output to repeated stimulation, which increases the flow of visual information toward the *Drosophila* brain. The present study was therefore aimed at understanding how network adaptation makes it possible to efficiently encode information. Through using synaptically altered flies (Fig. 9), we showed that feedforward and feedback pathways are necessary components for network adaptation, contributing to both “flattening” and “whitening” of LMC output. Most of this function can be removed by tampering with the feedforward, *e.g.* by *ort^6^* mutation, or the feedback pathways, *e.g.* by reducing synaptic transmission from L2 cells back to R1–R6 cells. The defective R-LMC pathway sped up the LMC and probably AC output, mimicking extreme light adaptation, while the defective L2-R pathway slowed down the LMC output, mimicking dark adaptation. This study, thus, further suggests that network adaptation plays an important role in matching the neural encoding strategies efficiently to environmental stimulus conditions.

### Network adaptation involves both feedforward and feedback pathways

Voltage responses in LMCs with genetically altered synapses lack adaptation and low frequency (*ort^6^*; Fig. 2D) or high-frequency information (*L2-shi^TS1^* at 30°C; Figs. 7C and 8D). What do these differences tell about the mechanisms of network adaptation in WT flies?

Results from *ort^6^* flies (Fig. 2) strongly suggest that their mutated receptors are largely desensitized during naturalistic stimulation and their low affinity for histamine [8] makes removal of desensitization, *i.e.* return of sensitivity, difficult. When only vigorous changes in histamine input remove desensitization and briefly reopen the channels, *ort^6^* LMCs integrate naturalistic stimuli inefficiently, generating fast responses at all luminance levels (Fig. 2D). Here, the reduced histamine-sensitivity of ort^6^ receptors [8], [10] further depolarizes LMCs [3] and likely ACs, increasing their excitatory feedback to R1–R6. Thus, even dark-adapted photoreceptor terminals can be driven toward light-adapted potentials [3]. Such paradoxical extreme “light adaptation” reduces valuable redundancies in naturalistic stimuli, preventing *ort^6^* synapses from improving their transmission by previous experience. Such “light adaptation” is particularly detrimental at dim stimulation (low signal-to-noise ratio) where the best coding strategy is to amplify slow visual events [3], [21]. Failure to adapt therefore compromises the quality of neural images for all but extremely bright stimulation (Fig. 2A) [3].

WT LMCs and LMCs from genetic controls for *L2-shi^TS1^*, in contrast, adapt appropriately at different luminance levels [4]. The first response is always desensitized (*cf.* Fig. 5A), but with repetitive stimulation the sensitivity gradually returns, improving the amplitude and frequency spectra (Fig. 3D; dotted lines) of the responses [4]. As this happens at different rates at different luminance levels, the findings imply that adaptation is also coupled to the rate of histamine release [4].

Recordings from *L2-shi^TS1^* flies at 19 and 30°C (Figs. 4 and 6) verify the role of L2-R feedback in network adaptation. Reducing this feedback removes an important sensitivity control mechanism [3], reducing and slowing down the synaptic signal transfer from R1–R6 photoreceptors to LMCs. Therefore, unlike with mutated histamine receptors, responses of LMCs with impaired L2-R feedback fail to gather relevant high-frequency information in naturalistic stimuli (Figs. 7C–D and 8D), but again we see little adaptive trends in the LMC output (Figs. 6C–D).

Taken together (Fig. 9), these findings suggest that the dynamically adjusted equilibrium between the light-mediated conductance, *i.e.* histamine-gated Cl^−^conductance, and feedback-mediated synaptic conductances [11], [12] in WT flies could facilitate matching the coding strategy of the retinal network to the ambient illumination. The findings further suggest that the probability of histamine release from photoreceptors could be dynamically matched to the opening probability of histamine-channels in LMCs so that the transmitter can deliver the message as best intended [21], [22] at different luminance levels.

We have considered the mechanisms of network adaptation in our experimental paradigms independent of high-order processing in the fly brain. However, it is probable that the tendency of flies to view “interesting” objects [23], [24], *i.e.* top-down regulation, also affects the throughput of the first visual synapses. Every synaptic layer from the eyes to the brain contains extensive feedback connections. For example, the next processing layer, the medulla, is linked retinotopically to the lamina by C2–C3 fibers [1], [11], [12], and we expect that these and other circuits in the medulla [25], [26] are also participating in the early processing of visual information.

### Role of other synaptic connections in early visual processing

Although L2-R feedback is not the only local feedback connection to R1–R6 photoreceptors, disrupting it greatly lowers network adaptation. What roles does this leave for amacrine and L4 cells [1] in local information processing?

Amacrine cells, which share the histamine receptors of LMCs, should hyperpolarize to light increments and contribute in the sensitivity control of R-LMC synapses [3], [27]. In addition, lateral branches of amacrine cells and L4 cells connect neighboring processing units [28]. Thus, they should mediate interactions between ommatidia that represent different points of the visual field. Unfortunately, we still lack direct evidence for these ideas.

Nevertheless, all laminal cells participate in routing and processing information in a dynamic and homeostatic manner; individual sub-networks can extend their roles if their neighbors are damaged or silenced [3], [29]. For instance, signals cross the faulty *ort^6^* receptors, but only because the histamine release from photoreceptors is boosted by synaptic feedback. For similarities to the *Drosophila* neuromuscular junction, see [30]. Thus, when a cell is embedded in the many parallel processing tasks of a network, its coding may vary from one stimulus situation to another, or it may perform several coding functions in conjunction.

### Relating physiology to anatomy and visual behavior

This work has highlighted the benefits of *in vivo* physiology, *i.e.* live-monitoring of neural activity, for understanding the role of neural circuits in local information processing. If we cannot assess the natural flow of information within the circuits, it is difficult to understand their processing properties.

Recently, it was shown that the visual behavior of *Drosophila* with genetically-inactivated L1 or L2 monopolar cell pathways differed significantly, suggesting that these pathways have specific roles in motion detection [15]. However, the lack of physiological evidence leaves many questions open. For example, why is the L2 pathway more sensitive to pattern contrast and low light intensity than the L1 pathway?

Here, recordings from *L2-shi^TS1^* flies provide a possible answer. By compromising the L2 pathway, we also affect the L2-R feedback that directly modulates the neural function of the L1 pathway. Thus, when information flow is reduced in the L2-pathway, the speed of signaling in the L1 pathway should slow down dramatically. The L1 output would deteriorate simply because L2-R feedbacks can no longer rescale the frequency spectrum of R1–R6 output synapses, including the input to L1 (Fig. 7C). In contrast, we predict that when the L1 pathway is inactivated alone, the fly brain still receives high-precision frequency-modulated information via the L2 pathway. At dim or at low contrast conditions of low signal-to-noise ratio, these effects naturally impact the behavior of warm *L2-shi^TS1^* flies the most (*cf.* Fig. 8D).

Therefore, with the behavioral data alone, conclusions about network function are only suggestive; we also would interpret the data reported by Rister *et al.* [15] as showing that the L2 pathway is more sensitive for certain stimuli than the L1 pathway. Instead, physiological evidence at 30°C from *L2-shi^TS1^* flies clarify that L1s are likely to be underperforming, as their out-of-tune photoreceptor input (owing to altered/silenced L2-R feedback) lacks the relevant stimulus frequencies. Hence, at least in their input level, L1s and L2s are equally sensitive in healthy WT flies as they share the same number of synapses [6], [7].

## Materials and Methods

### Flies

Wild type (WT) Oregon-R and Canton-S strains, *ort^6^* flies (sometimes named *ort* ^P306^) [8], and *L2-shi^TS1^* flies, in which expression of a *shibire^TS1^* transgene [16] was driven using *L2-Gal4* (previously named *21D-Gal4*, targets L2 monopolar cells with relative selectivity) [15], [31] were used for recordings.

The *ort^6^* flies have Oregon-R background [32] and wild type eye pigmentation. The *L2-shi^TS1^* flies with insertions of P{w^+^, *UAS-shibire^TS1^*} and P{w^+^, *L2-Gal4*} on chromosomes two and three, respectively (*w* ^+^; *UAS-shibire^TS1^*/+; *L2-Gal4*/+), were generated (with their 1^st^ chromosome homozygous, and the 2^nd^ and 3^rd^ chromosomes heterozygous for our original Canton-S stock). To make this stock, we first crossed each insertion line to an “X-chromosome Canton-S double balancer stock” (*w* ^+^; *If/CyO*; *MKRS/TM6b*) over two generations to produce *w* ^+^; *UAS-shibire^TS1^/CyO*; *MKRS/TM6b* and *w* ^+^; *If/CyO*; *L2-Gal4/TM6b* stocks. These stocks were then crossed to each other to produce a balanced stock carrying both insertions (*w* ^+^; *UAS-shibire^TS1^/CyO*; *L2-Gal4/TM6b*), which was further crossed to our wild type Canton-S strain to produce w^+^; *UAS-shibire^TS1^*/+; *L2-Gal4*/+ progeny. *UAS-shibire* ^TS1^ and *L2-Gal4* control flies were made in a similar manner, first balancing with “Canton-S single balancer stocks”, which contain non-balancer chromosomes of Canton-S origin (*w* ^+^; *Gla/CyO*; + and *w* ^+^; +; *MKRS/TM6b*, respectively), and then crossing back to our Canton-S line to produce *w* ^+^; *UAS-shibire* ^TS1^/+; + and *w* ^+^; +; *L2-Gal4*/+.

All flies were reared on standard medium at 18°C in 12∶12 light∶dark cycle [32], [33] and females were selected for electrophysiological experiments 4 days after eclosion. These flies have fully matured visual systems; their photoreceptors and LMCs respond adequately to naturalistic stimulation (NS), for all the genetic backgrounds used. Control experiments for *ort^6^* and WT Oregon-R using younger and older flies (2–12 days old) [3] indicated that after 4 days recordings from mutant LMCs become more difficult.

### *In vivo* electrophysiology

Flies were prepared for *in vivo* experiments as in [2]. During the experiments, the flies were immobilized within a brass fly-holder, placed on a Peltier-device [18]. The ambient air temperature was maintained by air conditioning at 19.0±0.5°C, while the head-temperature of the flies was set to 19, 25 or 30±0.5°C through a feedback-controlled Peltier-device [18]. Because of the low success rate of stable recordings in small LMCs (>10 min) while warming or cooling the flies, *L2-shi^TS1^* experiments and their genetic controls were performed separately at 19°C and 30°C mostly using different flies. Acclimatization of over 5 min before the experiments ensured that the temperature-sensitive endocytotic *shibire^TS1^*-function should either block (30°C, restrictive temperature) or allow (19°C, permissive temperature) signaling [3] via L2-R feedback connections.

### Intracellular voltage responses and selection criteria

To prevent poor penetrations or electrodes biasing our analysis, only stable high quality recordings were used. Such photoreceptors had resting potentials in darkness <−60 mV and maximum responses to bright naturalistic stimulation >35 mV (WT Oregon-R, WT Canton-S, *ort^6^*, *L2-shi^TS1^*, *L2-Gal4*, *UAS-shi^TS1^*). For the selected LMCs, the resting potentials were <−30 mV and maximum responses >15 mV at 19–25°C (WT Oregon-R, WT Canton-S, *L2-shi^TS1^*, *L2-Gal4*, *UAS-shi^TS1^*) or >10 mV (*ort^6^*). At >30°C, responses of LMCs are slightly smaller, because of faster signaling (Fig. 5A–B). Note that some LMCs included here had smaller amplitudes than in our previous study [3] because their response dynamics, when normalized, were similar to those of cells with the largest responses (30–50 mV). See Figures 5C–F, including signal-to-noise ratio in Figure 8.

### Light stimulation and data collection

These methods were identical to the ones described in the companion paper [4]. In brief: naturalistic light intensity time series from the van Hateren natural-stimulus-collection, http://hlab.phys.rug.nl/archive.html [34] were use to drive a LED. The light stimulus was delivered to the fly eye by a randomized fiber optic bundle, secured on a Cardan arm system. The light stimulus subtended 5°, as seen by the fly, and its brightness was controlled in an intensity range that covered 4 log-units [3], [19] from ∼600 to ∼6×10^6^ photons/s (*I*_0_). Figures show results for dim- (1,850), medium- (60,000) and bright-light (1.85×10^6^ photons/s). Typically, the cells were first dark-adapted for 30 s and then presented with dim stimulation before brightening the stimulation. Between luminance levels the cells were re-dark-adapted for 30 s. The stimulus and response were sampled at 10 kHz.

### Probability density and joint probability functions

The probability density (PDF) and joint probability density (jPDF) functions of the photoreceptor and LMC outputs to the repeated stimulation were measured by mapping one-to-one their corresponding voltage values at 1 ms time-resolution over the evolution of the experiments as explained in the part I [4].

### Adaptive trends in continuous recordings

We quantified adaptive trends in a cell's responsiveness to the same repeated stimulus pattern by taking the standard deviation (SD) of the first 20 voltage responses, using the last 8,000 samples (time points) of each response as explained in the part I [4]. In general, SD was used as a simple metric to track adaptive changes in photoreceptor and LMC outputs, since we have shown that their wider distributions equal greater sensitivity [4].

In total, ∼83% of all the recorded WT LMC outputs (180/218) to repetitive naturalistic stimulation showed adaptational increase in sensitivity at the preferred temperatures (19–25°C) [35]. Thus, only ∼17% of the LMCs showed no trends or decreasing sensitivity; some of which we assessed to be of good quality. This suggests that either one LMC subgroup (L1–L3) or AC may adapt differently. Note, if these cells occupied equal volumes (untrue) they would share the same probability of being penetrated (P = 0.25). However, it is more likely that ∼2/3 of the recordings were from the largest cells (L1 and L2), both of which therefore must show adaptational increase in sensitivity to repeated stimuli. It is also our experience through electrophoretically staining *Calliphora* LMCs that most recordings are from L1–L2 cells, but that L3 response waveform to light is relatively similar to those of L1–L2s [6]. See also [5].

### Frequency Analysis

We quantified changes in photoreceptor and LMC output in frequency domain by comparing corresponding frequency spectra (spectra) of the photoreceptor and LMC outputs in WT and mutant flies at each second of repetitive stimulation. Matlab's Fast Fourier Transform (FFT) algorithm was used to calculate the power spectra 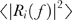 for each 1 s long response (after resampling at 1 kHz) [4]. The square root of the power spectra then gave the mean frequency spectrum 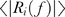 for each response to the repeated stimulus (Fig. 7).

The mean frequency spectra for each luminance level were averaged (Fig. 3B). The ratio between the corresponding mean photoreceptor and LMC frequency spectra gave the synaptic gain (Fig. 3) function for each stimulus presentation or for each light level.

Signal-to-noise ratio (Fig. 8), SNR(*f*), is the ratio between the signal 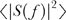 and noise 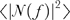 power spectra. The signal power spectrum was calculated from the mean voltage response, using 10–20^th^ s responses to the same stimulus pattern. At this point most of the adapting trends have already subsided as the responsiveness approaches relative stationarity (*cf.* Fig. 6C). The noise power spectra were calculated from the corresponding noise traces, *i.e.* the differences between individual responses and signal. The data chunks were divided into 50% overlapping stretches and windowed with a Blackman-Harris 4-term window, each giving three 500-points long samples. These were then averaged to improve the estimates. More details are given in [2].

## Supporting Information

Figure S1 Reducing L2-R-feedback reduces the high-frequency content and hence adaptational “whitening” of LMC output. A. Mean output of WT Canton-S LMCs to middle-intensity naturalistic stimulus (NS) at 19 oC (left) and 30 oC (right), n = 13 and 14 cells, respectively. B. The mean normalized spectra of LMCs (n = 11–14 cells) measured at the 20th s of NS in WT (black), *L2-shi^TS1^* (red), *UAS-shi^TS1^* (blue) and *Gal4-L2* flies (violet) at 19 oC (left) and 30 oC (right). At 19 oC, these frequency distributions are practically identical. At 30 oC, *L2-shi^TS1^* LMCs show a reduced high-frequency content. Subfigures C–F compare the mean spectra of these LMCs to the 1st and 20th s of middle-intensity NS at 19 oC (left) and 30 oC (right). At 19 oC, the spectra show the “whitening” effect of synaptic adaptation; the middle- and high-frequency content of the stimulus being boosted mostly over time. At 30 oC, because of the temperature-dependent quickening of bio-reactions, the frequency distributions of LMCs have increased high-frequency contents. Here, all but *L2-shi^TS1^* LMCs (gray background) showed similar adaptational “whitening” as occurred at 19 oC, indicating that shibireTS1 had selectively reduced communication from L2-cells back to R1–R6 photoreceptors. This data, thus, highlights the role of L2-R-feedback in the frequency-dependent sensitivity control of LMC output. With compromised L2-R feedback, LMC output slows down (reduced high-frequency range) and fails to optimize the neural representation of the dynamic light contrast changes for the brain.
(1.26 MB TIF)
