# Supplementary figures and images for: Network Adaptation Improves Temporal Representation of Naturalistic Stimuli in Drosophila Eye: II Mechanisms
Source: PLoS One. 2009 Jan 30;4(1):e4306. doi: 10.1371/journal.pone.0004306 (PMC2628722; doi:10.1371/journal.pone.0004306)

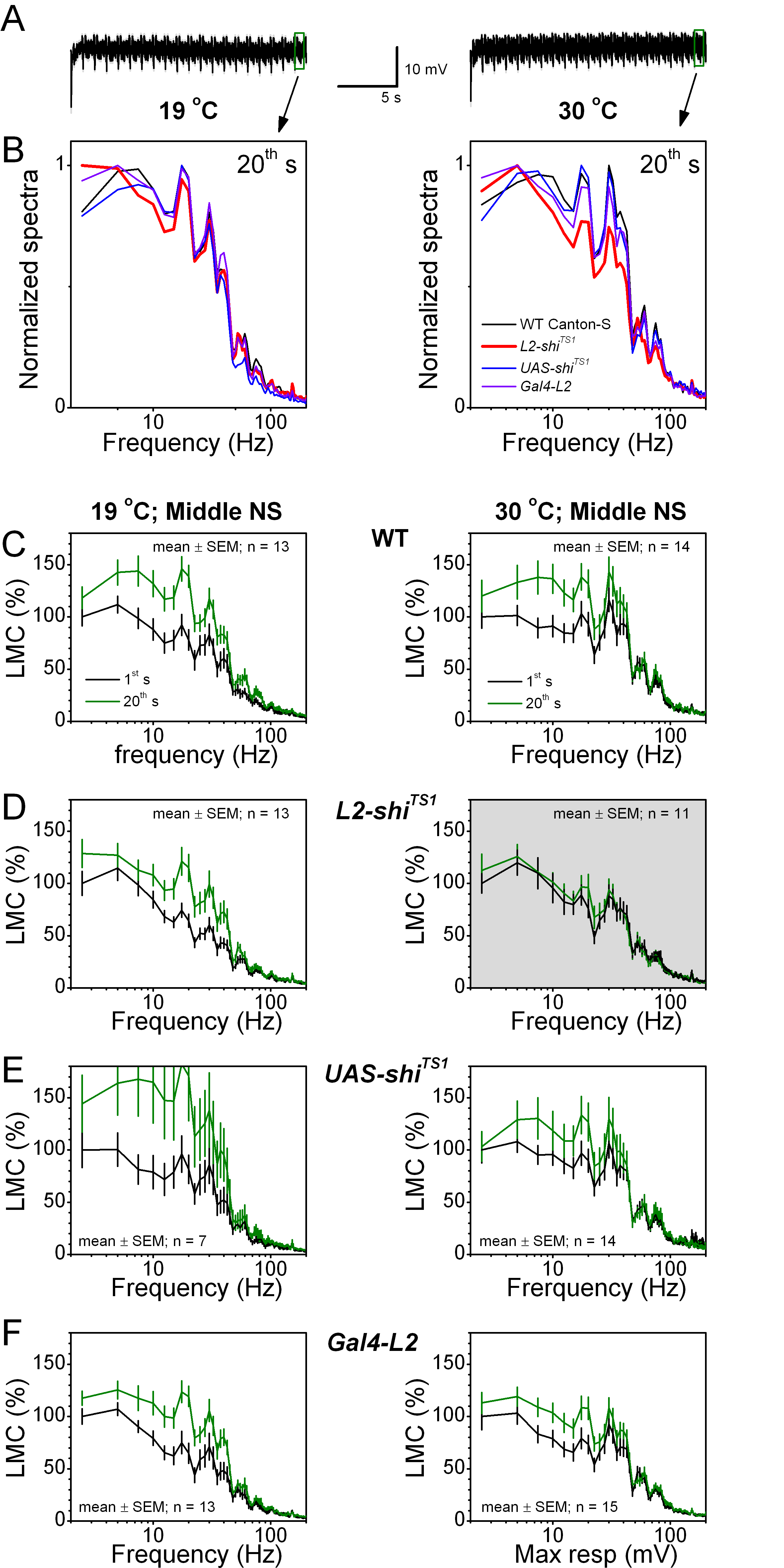

Supplement: Figure S1 — Reducing L2-R-feedback reduces the high-frequency content and hence adaptational “whitening” of LMC output. A. Mean output of WT Canton-S LMCs to middle-intensity naturalistic stimulus (NS) at 19 oC (left) and 30 oC (right), n = 13 and 14 cells, respectively. B. The mean normalized spectra of LMCs (n = 11–14 cells) measured at the 20th s of NS in WT (black), L2-shiTS1 (red), UAS-shiTS1 (blue) and Gal4-L2 flies (violet) at 19 oC (left) and 30 oC (right). At 19 oC, these frequency distributions are practically identical. At 30 oC, L2-shiTS1 LMCs show a reduced high-frequency content. Subfigures C–F compare the mean spectra of these LMCs to the 1st and 20th s of middle-intensity NS at 19 oC (left) and 30 oC (right). At 19 oC, the spectra show the “whitening” effect of synaptic adaptation; the middle- and high-frequency content of the stimulus being boosted mostly over time. At 30 oC, because of the temperature-dependent quickening of bio-reactions, the frequency distributions of LMCs have increased high-frequency contents. Here, all but L2-shiTS1 LMCs (gray background) showed similar adaptational “whitening” as occurred at 19 oC, indicating that shibireTS1 had selectively reduced communication from L2-cells back to R1–R6 photoreceptors. This data, thus, highlights the role of L2-R-feedback in the frequency-dependent sensitivity control of LMC output. With compromised L2-R feedback, LMC output slows down (reduced high-frequency range) and fails to optimize the neural representation of the dynamic light contrast changes for the brain. (1.26 MB TIF) [file pone.0004306.s001.tif]
